# Supplementary material for: Splice-Junction-Based Mapping of Alternative Isoforms in the Human Proteome
Source: Cell Rep. Author manuscript; Available in PMC 2020 Jan 15. (PMC6961840; doi:10.1016/j.celrep.2019.11.026)
Supplement: 2 [file NIHMS1546469-supplement-2.zip › DF1/PXD009737/TestisTrypsin/TestisTrypsin_33_DPP7_RPPAHSPVPAPPLR.pdf]

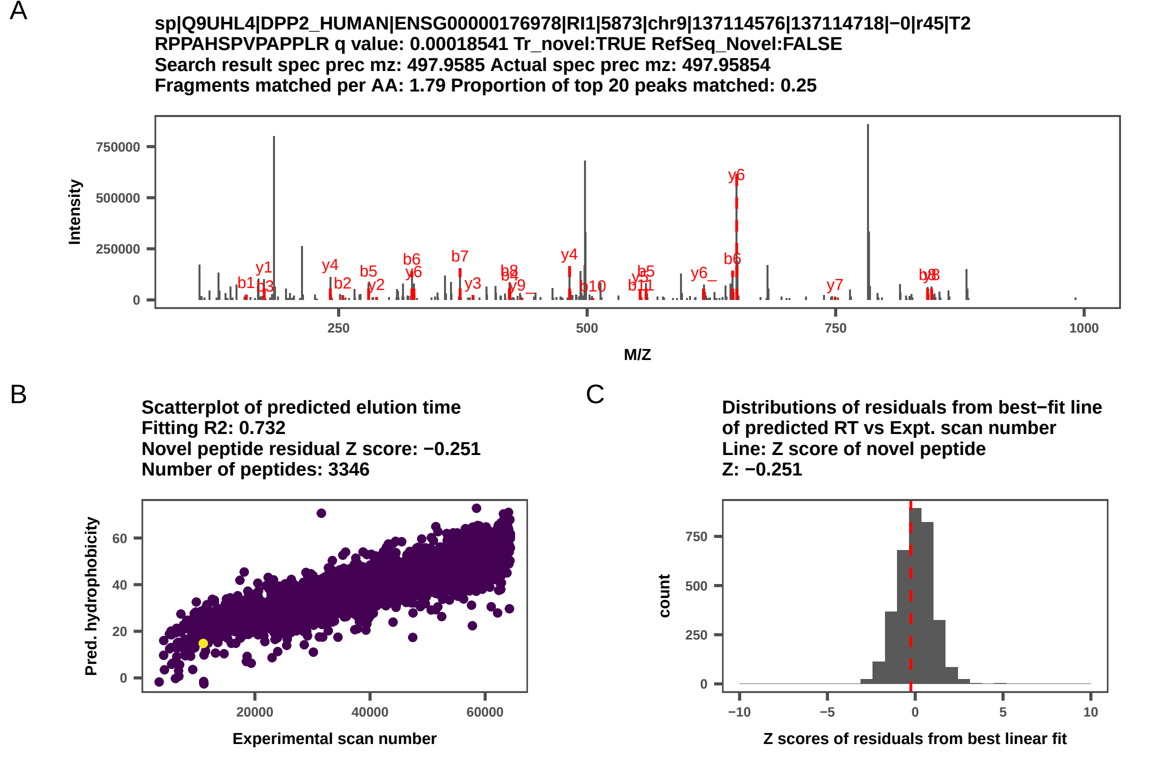
A
sp|Q9UHL4|DPP2_HUMAN|ENSG00000176978|RI1|5873|chr9|137114576|137114718|−0|r45|T2
RPPAHSPVPAPPLR q value: 0.00018541 Tr_novel:TRUE RefSeq_Novel:FALSE
Search result spec prec mz: 497.9585 Actual spec prec mz: 497.95854
Fragments matched per AA: 1.79 Proportion of top 20 peaks matched: 0.25
Intensity
750000
500000
250000
0
250
500
750
1000
M/Z
B
Scatterplot of predicted elution time
Fitting R2: 0.732
Novel peptide residual Z score: −0.251
Number of peptides: 3346
Pred. hydrophobicity
60
40
20
0
20000
40000
60000
Experimental scan number
C
Distributions of residuals from best−fit line
of predicted RT vs Expt. scan number
Line: Z score of novel peptide
Z: −0.251
count
750
500
250
0
−10
−5
0
5
10
Z scores of residuals from best linear fit
